# Hypoxia Increases Mouse Satellite Cell Clone Proliferation Maintaining both *In Vitro* and *In Vivo* Heterogeneity and Myogenic Potential

**DOI:** 10.1371/journal.pone.0049860

**Published:** 2012-11-16

**Authors:** Luca Urbani, Martina Piccoli, Chiara Franzin, Michela Pozzobon, Paolo De Coppi

**Affiliations:** 1 Fondazione Città della Speranza, Monte di Malo, Vicenza, Italy; 2 Department of Woman and Child Health, University of Padova, Padova, Italy; 3 Surgery Unit, University College of London, Institute of Child Health, Great Ormond Street Hospital for Children, London, United Kingdom; University of Minnesota, United States of America

## Abstract

Satellite cells (SCs) are essential for postnatal muscle growth and regeneration, however, their expansion potential *in vitro* is limited. Recently, hypoxia has been used to enhance proliferative abilities *in vitro* of various primary cultures. Here, by isolating SCs from single mouse hindlimb skeletal myofibers, we were able to distinguish two subpopulations of clonally cultured SCs (Low Proliferative Clones - LPC - and High Proliferative Clones - HPC), which, as shown in rat skeletal muscle, were present at a fixed proportion. In addition, culturing LPC and HPC at a low level of oxygen we observed a two fold increased proliferation both for LPC and HPC. LPC showed higher myogenic regulatory factor (MRF) expression than HPC, particularly under the hypoxic condition. Notably, a different myogenic potential between LPC and HPC was retained *in vivo*: green fluorescent protein (GFP)+LPC transplantation in cardiotoxin-injured *Tibialis Anterior* led to a higher number of new GFP+muscle fibers *per* transplanted cell than GFP+HPC. Interestingly, the *in vivo* myogenic potential of a single cell from an LPC is similar if cultured both in normoxia and hypoxia. Therefore, starting from a single satellite cell, hypoxia allows a larger expansion of LPC than normal O_2_ conditions, obtaining a consistent amount of cells for transplantation, but maintaining their myogenic regeneration potential.

## Introduction

Adult skeletal muscle exhibits a considerable capacity to regenerate and completely repair its structure and physiological function quickly after injury. During postnatal development, new myonuclei are supplied by muscle-specific stem cells, known as satellite cells (SCs), which divide to provide myoblasts for muscle growth and/or repair [1], [2]. Anatomically identified as mononuclear cells that reside between the sarcolemma and the surrounding basal lamina of individual myofibers [3], these muscle-specific stem cells are normally in an undifferentiated mitotically-quiescent state within the sublaminal niche [1], [4], but they are activated in response to stress, induced by growth or trauma. Activated SCs, called myogenic precursor cells, proliferate and differentiate to fuse with each other or with existing multinucleated myofibers [5]. Moreover, they can generate progeny that re-establish the quiescent SCs pool, a self-renewing process essential for sustaining the SC compartment [6].

The stem cell niche is a three dimensional informative structure that physically defines stem cells’ localization and directs them to activation, differentiation and self-renewal. The balancing between signals required for stem cell quiescence or activation is the key homeostatic function of the stem cell niche. This dynamic microenvironment spatially and temporally controls stem cells by a mixture of chemokines, cytokines, extracellular matrix molecules and gases. The understanding and mimicking of this complex niche is extremely important for a more thorough stem cell characterisation and culture. Indeed, stem cell cultures usually show limited self-renewal or expansion, suggesting that classical stem cell culture conditions need to be optimized. Reproducing *in vitro* the stem cell microenvironment can be complex but the benefits would include a better understanding of stem cell biology and expansion for regenerative medicine prospective. Among the different factors influencing the niche, oxygen concentration plays a particularly relevant role in various tissues. Increasing evidence demonstrates that oxygen tension does not represent only a metabolic substrate, but also an important signalling molecule within the stem cell niche, where it regulates cell proliferation and plasticity [7]–[10]. Stem cell responses to different oxygen tensions have been studied in several cell types, such as neural stem cells, haematopoietic stem cells and, more recently, human SCs [8]–[14]. In these studies, hypoxic conditions produced an enhancement of stem cell proliferation and a modification of the cell fate when induced to differentiate. Physiological oxygen tension in the majority of tissues is limited and, generally, lower than classic culture conditions (20% O_2_). In particular, in the adult skeletal muscle, oxygen levels in physiological conditions generally vary between 2% and 10%, depending on the analysed muscle [7], [15]. Indeed, *in vivo* hypoxic condition (ischemic muscle damage) produces SC activation, proliferation, differentiation and maturation to muscle fibers, as well as maintaining the local stem cell pool [11], [16], [17]. Other then oxygen (low or high) stimulus, activation of SCs leading to myogenesis is regulated by transcriptional and epigenetical control of myogenic regulatory factors (MRFs), which comprise of myogenic factor 5 (Myf5) and myoblast determination protein (MyoD) [18]–[20]. While quiescent SCs express the paired/homeodomain gene *Pax7*, satellite cell-derived myoblasts firstly proliferate and express both *Pax7* and *MyoD*, then downregulate *Pax7* maintaining *MyoD* expression throughout the myogenic differentiation process. A population of Pax7+MyoD+cells, however, withdraw from the cell cycle, downregulate *MyoD* and return to quiescence by sustaining *Pax7* expression [21], through a self-renewal process.

**Figure 1:**
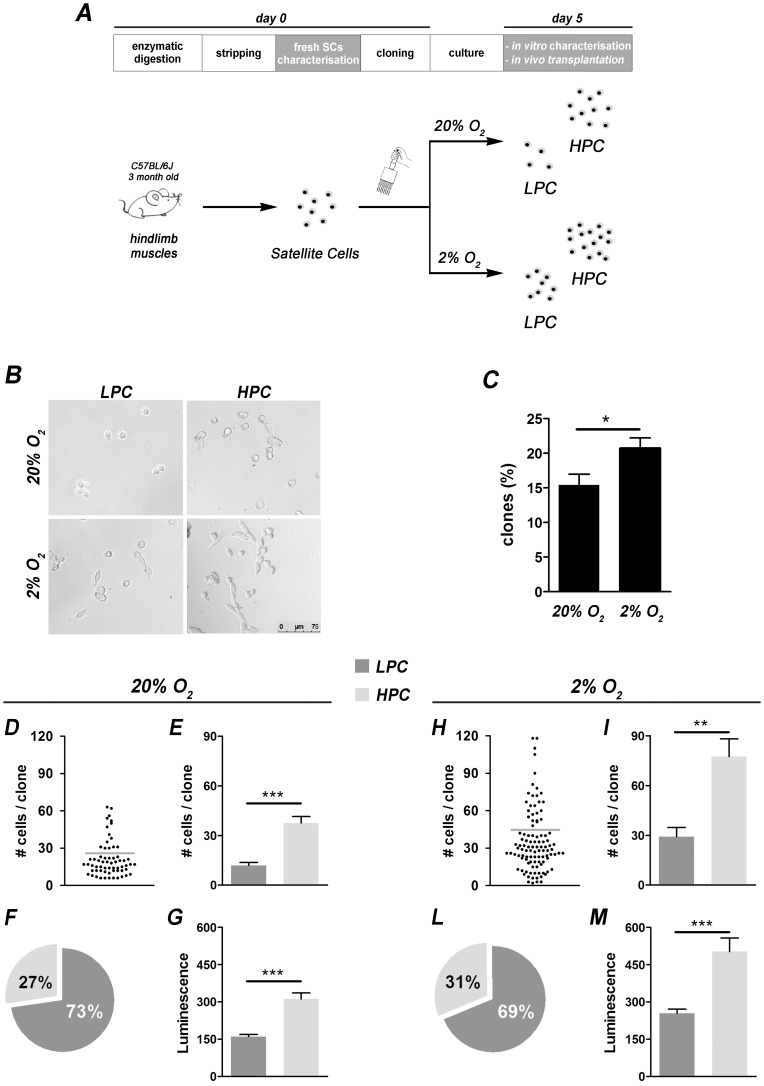
Mouse satellite cell isolation, cloning and *in vitro* proliferation. (A) Experimental plan**.** For satellite cell (SC) isolation, hindlimb skeletal muscles of C57BL/6J mice were digested and single myofibers collected. SCs were mechanically disengaged (stripping passage) and cloned with the limited dilution method. For each experiment, cloned cells were randomly cultured in normoxic (20% O_2_) or hypoxic (2% O_2_) conditions with a proliferative medium. (B) After 5 days of culture, all the clones had a similar morphology (bar = 75 µm). (C) The number of clones generated in hypoxia was higher than in the normoxia conditions, expressed as the number of clones obtained starting from the same seeded cell number (n = 16, mean±SEM, * p<0.05). (D and H) Number of cells *per* clone distribution (example experiment) under normoxia or hypoxia conditions respectively (a model based cluster analysis with normal mixture modelling has been applied to identify the groups): cells distribute in 2 clusters easily separable with a cut-off value (light grey bar) in LPC and HPC. (E and I) Averages of the number of cells *per* clone both in LPC and HPC demonstrated that the proliferation increases in those clones cultured with 2% O_2_ (total number of clones counted: 20% O_2_ n = 600, 2% O_2_ n = 700, mean±SEM, ** p<0.01, *** p<0.001). (F and L) Pie charts indicate LPC and HPC proportions calculated as percentage of the total number of clones (n = 16, mean±s.d., 20% O_2_: LPC = 73%±8.1, HPC = 27%±8.1; 2% O_2_: LPC = 69%±8.3, HPC = 31%±8.3; p<0.001). (G and M) ATP quantification: LPC show lower ATP content with respect to HPC, both in normoxia and hypoxia. ATP measurements uphold the higher number of cells both in LPC and HPC under hypoxia (n = 132, mean±SEM, *** p<0.001).

*In vivo* grafting experiments showed the ability of SCs to give rise to both new myofibers and new SCs maintaining the stem cell pool throughout life: for this reason, SCs are considered the natural source for muscle tissue-engineering. However, accounting for only 2–4% of the total myofiber nuclei [22], achievement of sufficient numbers of cells for transplantation is essential for clinical application. Consequently, more attention has been recently directed to improving the expansion of SCs before *in vivo* application, maintaining their myogenic potential and regenerative capacity. However, in order to obtain the best results, the *in vitro* culture of SCs should mimic the *in vivo* microenvironment, providing some stimuli that are present in the specific niche in which SCs normally reside.

Given the sensitivity of SCs to their physiological microenvironment and that they react to *in vivo* hypoxic muscle conditions with activation, proliferation and differentiation, also maintaining their stem cell reserve pool, we hypothesized that culturing SCs at a low level of oxygen, such as in their native niche, could give insight into cells’ heterogeneity and, at the same time, more expansion potential. Here we aimed to compare the effect of hypoxic (2% O_2_) vs. traditional culture conditions (normoxia –20% O_2_) on the heterogeneity, clonogenicity, proliferation, marker expression, differentiation and *in vivo* potential of mouse SC clones from freshly isolated myofibers. As in our previous experiments in rats [23], after 5 days of culture it was possible to distinguish two subpopulations of mouse SC clones: low proliferative clones (LPC) and high proliferative clones (HPC) with distinct proliferation rates. Hypoxia increased the proliferation of all the clones, but maintained the distinction and the proportion between the two subpopulations. LPC also revealed higher amounts of MRFs and CD34 expression. Most importantly, single LPC transplanted in cardiotoxin-injured muscles revealed stronger engraftment and muscle regeneration than HPC. Notably, the Engraftment Index calculated by a single LPC cell was similar as it has been cultured under 2% or 20% of O_2_, proving that hypoxia led to the expansion of SCs, maintaining their intrinsic heterogeneity without loosing their myogenic capacity.

**Figure 2:**
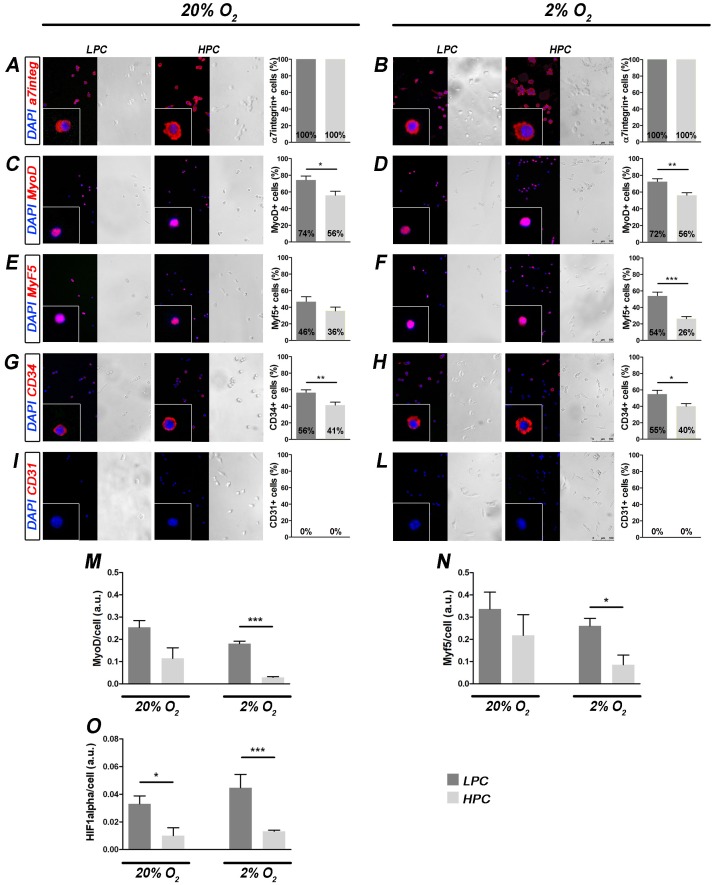
Marker expression in SC clones cultured under normoxia and hypoxia. Immunofluorescence analyses of LPC and HPC cultured for 5 days at 20% and 2% O_2_ revealed differences in the expression of MyoD, Myf5 (only at 2% O_2_) and CD34. Phase-contrast and corresponding immunostaining examples for the specific marker (in red) merged with DAPI (left, scale bar = 100 µm, insets with higher magnification). Graphs indicate the percentage of marker-positive cells *per* clone (right, mean±SEM, * p<0.05, ** p<0.01, *** p<0.001): α7integrin (A) and (B) (n = 50), MyoD (C) and (D) (n = 70), Myf5 (E) and (F) (n = 85), CD34 (G) and (H) (n = 50), CD31 (I) and (L) (n = 45). (M, N and O) *MyoD*, *Myf5* and *Hif-1α* gene expression quantification was performed using RealTime PCR. Graphs indicate the amount of *MyoD*, *Myf5* and *Hif-1α* expression respectively at single cell level (n = 4, mean±SEM, ANOVA analysis determined a significant difference among groups for *MyoD* and *Hif-1α* expression; Student t-test: * p<0.05, *** p<0.001).

**Figure 3:**
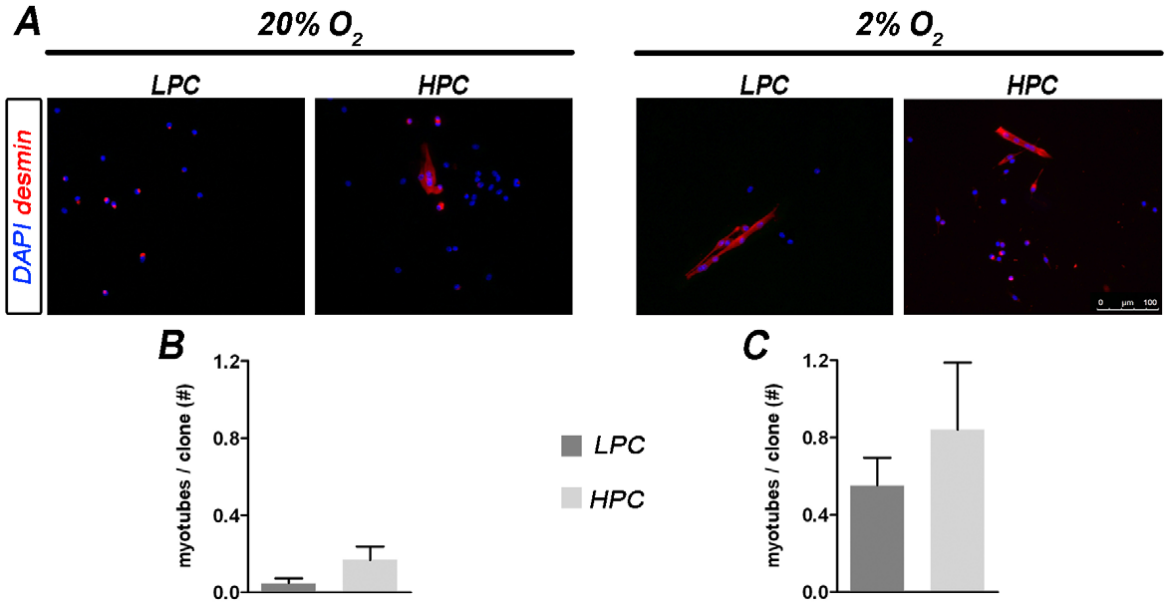
Spontaneous myogenic differentiation in LPC and HPC at day 5 of culture. (A) Spontaneous myotubes formation after 5 days of culture in proliferative medium was evaluated with desmin staining (in red). Immunofluorescence shows the appearance of single desmin+cells in LPC cultured in 20% O_2_ and also some myotube formation in HPC at 20% O_2_ and both in LPC and HPC cultured at 2% (bar = 100 µm, nuclei counterstained with DAPI). (B and C) Graphs indicate the number of myotubes *per* clone calculated for all the clone types (LPC 20% O_2_ n = 390, HPC 20% O_2_ n = 205, LPC 2% O_2_ n = 425, HPC 2% O_2_ n = 265; mean±SEM). Both LPC and HPC showed increased myotube formation when cultured at 2% O_2_ (ANOVA analysis determined a statistical difference among the groups; Student t-test: p<0.05 for number of myotube/clone in hypoxia cultured clones with respect to both clone types cultured under normoxia).

## Methods

### Animals

Three month-old male wild-type C57BL/6J mice and transgenic C57BL/6-(ACTB-EGFP)/J mice, with the expression of the enhanced GFP under the control of chicken beta actin promoter, were used in this study.

Animal care and experimental procedures were approved by the University of Padua’s Animal care and Use Committee (CEASA, protocol #41056, 13/01/2010) and were communicated to the Ministry of Health and local authorities in accordance with Italian law.

### Single Myofiber Isolation

Single muscle fibers with associated SCs were isolated from hindlimb skeletal muscles after enzymatic digestion with 0.2% (w/v) type I-collagenase (Sigma-Aldrich), reconstituted in Dulbecco’s Modified Eagle Medium (DMEM) low-glucose (GIBCO-Invitrogen) (supplemented with 1% penicillin-streptomycin, GIBCO-Invitrogen) for 2 hours at 37°C as previously described [23]. Briefly, following digestion, muscles were transferred to a 100 mm horse serum-coated plate containing 9 mL of plating medium (1^st^ dilution), composed of DMEM low-glucose, 10% Horse Serum (HS, GIBCO-Invitrogen), 1% penicillin-streptomycin (P/S), 0.5% chicken embryo extract (CEE, Seralab) and gently stirred with the same medium using a micropipette to release single myofibers. To minimize the contribution of non-myogenic cells, under phase contrast microscope, single fibers were carefully sucked up and serially transfer through two 10 mm HS-coated dishes containing 9 mL of plating medium (2^nd^ and 3^rd^ dilution).

**Figure 4:**
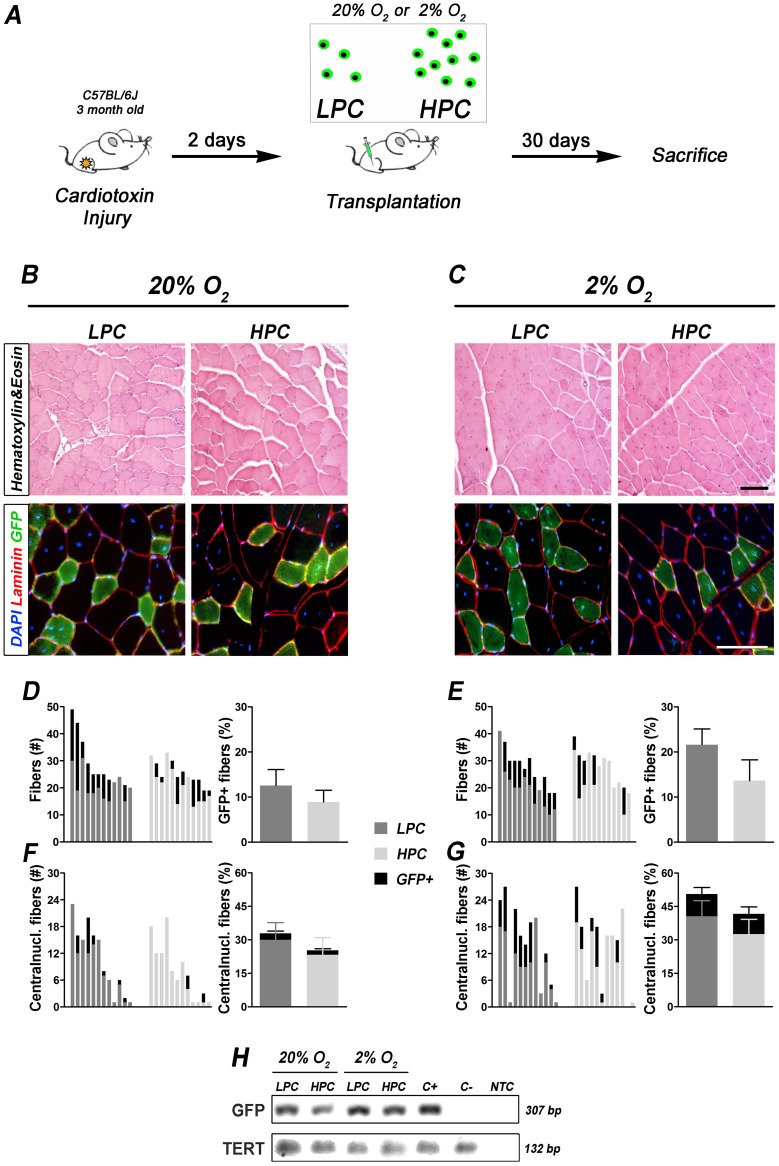
*In vivo* transplantation of LPC and HPC in injured muscles. (A) Experimental plan: single GFP+LPC and HPC were transplanted in *Tibialis Anterior* 2 days after cardiotoxin injury and muscle sections were analysed 30 days after cell injection. (B and C) GFP+LPC and HPC cultured at 20% and 2% O_2_ transplanted in damaged muscles. Haematoxylin and Eosin staining revealed a physiological muscle structure in both conditions 1 month post injection (upper row, bar = 100 µm). GFP+fibers were present in both groups with an increased presence in the LPC treated group in respect to HPC (staining for Laminin and GFP on transplanted TA sections, nuclei were counterstained with DAPI; lower row, bar = 100 µm). (D–G) Graphs of row numbers and percentages of GFP+fibers and GFP+centrally nucleated fibers in LPC and HPC 20% or 2% O_2_ treated muscles. (D and E) On the left, number of total fibers (gray: GFP- fibers, black: GFP+fibers *per* field) in representative fields of transplanted muscle sections. On the right, average of the percentages of GFP+fibers. (F and G) On the left, number of centrally nucleated fibers (gray: GFP- fibers, black: GFP+fibers *per* field) in randomly selected representative fields of transplanted muscle sections. On the right, average of the percentages of GFP+and GFP- centrally nucleated fibers (LPC 20% O_2_ n = 12, HPC 20% O_2_ n = 10, LPC 2% O_2_ n = 11, HPC 2% O_2_ n = 8). TA injected with normoxia cultured LPC possessed one third more green fibers than HPC (D) while GFP+centrally nucleated fibers were found in the same proportion in both treated animal groups (F). For hypoxia cultured clones, muscles LPC injected possessed about twice the number of green fibers than HPC (E). Centrally nucleated fibers were found in the same proportion in both treated animal groups but the TA treated with clones cultured under 2% O_2_ highlighted more centrally nucleated fibers than those transplanted with normoxia cultured clones (G) (no statistical differences between 20% and 2% O_2_ results). (H) PCR analysis of transplanted TA with GFP+single clones. First lane: amplification of the *GFP* gene (307 bp). Second lane: amplification of genomic *TERT* (132 bp) to confirm the quality of the extracted DNA. For each PCR, the positive control (C+) for GFP amplification was the genomic DNA extracted from C57BL/6-(ACTB-EGFP)/J mice muscle (NTC = no template control). In all samples GFP protein was detected.

**Figure 5:**
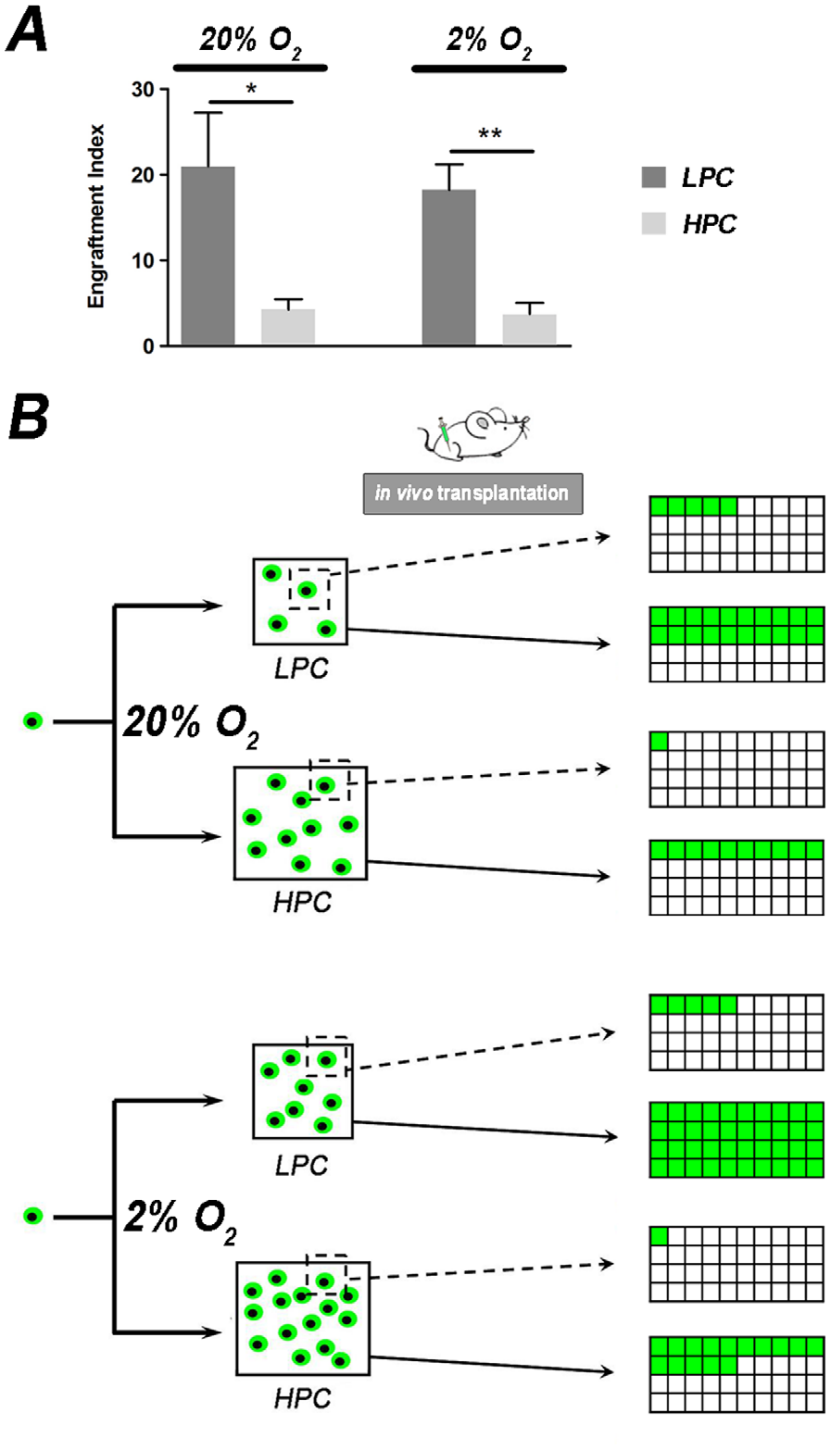
*In vivo* engraftment potential of LPC and HPC single cells. (A) Graphs with the Engraftment Index average calculated as proportion between GFP+fibers percentage and number of GFP+transplanted cells (for raw data see Table S2). In both normoxia and hypoxia conditions cells from LPC possess stronger myogenic potential (mean±SEM, p<0.01 with ANOVA, * p<0.05, ** p<0.01 with Student t-test). (B) Cartoon emphasizing on one hand the same myogenic potential at single cell level (dotted line) for LPC and HPC respectively when cultured at different conditions and, on the other (continuous line), the greater muscle regeneration ability of LPC cultured under 2% O_2_. In this condition, LPC proliferation is enhanced and as a consequence the achievable myogenic engraftment is higher.

### Primary Satellite Cell Isolation

Single isolated myofibers were transferred from the 3^rd^ dilution into a vial containing 1 mL of DMEM low-glucose. They were then triturated 20 times using an 18 G needle mounted onto a 1 mL syringe, to disengage SCs. The resulting cell suspension was filtered through 20 µm and 50 µm cell strainer and centrifuged at 1200 g for 5 minutes. The supernatant was gently removed and cells (named ‘stripped’ SCs) were suspended in phosphate buffered saline 1X (PBS) for citospun deposition or in a proliferative medium, composed of DMEM low-glucose with 20% Fetal Bovine Serum (FBS, GIBCO-Invitrogen), 10% HS, 1% P/S and 0,5% CEE, for clonal cultures.

Alternatively, for some experiments SCs were isolated from skeletal muscles through a FACS-sorting method [24]. In brief, we carefully removed nerves, blood vessels, tendons and fat tissue from hindlimb muscles under a dissection microscope. Muscles were minced and then digested with dispase (BD), collagenase type I (Sigma) and CaCl_2_ (Sigma) for 1 hour. Muscle slurries were filtered through a 70 µm and 100 µm cell strainer. The resulting freshly isolated mononuclear cell population was suspended in PBS containing 1% FBS and stained with anti SM/C-2.6 antibody (kindly provided by Fukada S.) for 30 minutes at 4°C [25]. Cy5-PerCp was used as the secondary antibody. Anti-CD45 PE, anti-CD31 PE and anti-Sca1 PE antibodies were used for lineage depletion. Cell sorting was performed on a FACSAria (BD Biosciences). The resulting sorted cell population was suspended in the proliferative medium for clonal cultures.

### The Cytospun of Primary Satellite Cells

Freshly isolated SCs obtained with the stripping method were suspended in PBS and were induced to adhere to glass coverslips (100 cells/coverslip) by centrifugation with cytospin Centrifuge MPW-223-c at 700 rpm for 5 minutes. Cell spots were then air-dried and fixed in 4% paraformaldehyde (PFA, Sigma-Aldrich) in PBS to be immunostained.

### Primary Satellite Cell Clonal Cultures

For each experiment, stripped SCs or SM/C-2.6+cells suspended in proliferating medium were dispensed into 96-well plates with limiting dilution (0.5 cell/well) (0.1 mL/well). Dishes were split into either 20% or 2% O_2_ conditions, both under 37.5°C and 5% CO_2_ in a humidified culture incubator. Clones were observed at 5 days and the cells were counted with inverted-microscope analysis.

### ATP Quantification

Cell viability quantification was performed using the CellTiter-Glo® Luminescent Cell Viability Assay (Promega) based on quantification of the ATP present, following manufacturer’s instructions. In brief, after 5 days in culture, clone plates were equilibrated at room temperature for 30 minutes and cell lysis was induced. By the addition of the specific CellTiter-Glo® Reagent, a luminescent signal was generated proportional to the amount of ATP. Luminescence was recorded with a luminometer (Packard Instruments).

### TUNEL Assay

Cell apoptosis was determined performing TUNEL assay as previously described [26] following manufacturer’s instructions. In brief, cells were permeabilized with 0.1% Triton X-100 (Sigma) on ice, washed twice with PBS and then incubated with a mixture of terminal deoxynucleotidyl transferase and fluorescein-labeled nucleotides for 1 h at 37°C. The total number of nuclei was visualized by fluorescence microscopy after incubation of cultures with DAPI for 10 min at room temperature. The apoptotic rate (percent of TUNEL-positive cells) was estimated by counting random fields for each clone.

### Immunocytochemistry on Freshly Isolated Satellite Cells

Citospuned stripped SCs were rinsed in PBS and permeabilized with Triton X-100 (Fluka) 0.5% in PBS for 5 minutes. Non-specific interactions were blocked with 10% HS for 30 minutes. After washing, cells were incubated with primary antibodies 1 hour at 37°C or overnight at 4°C. They were then washed and incubated with labeled secondary antibodies for 1 hour at 37°C. Nuclei were counterstained with fluorescent mounting medium (DAKO) plus 100 ng/mL 4′,6-diamidino-2-phenylindole (DAPI). The following primary antibodies were used: mouse anti-mouse Pax7 (Developmental Studies Hybridoma Bank, dil. 1∶20), rabbit anti-mouse Myf5 (Santa Cruz Biotechnology, dilution 1∶20), rabbit anti-mouse MyoD (Santa Cruz, dil. 1∶20), rat anti-mouse CD34 (GeneTex, dil. 1∶100), mouse anti-mouse α7integrin (dil. 1∶100), rat anti-mouse CD31 (eBioscence, dil. 1∶80), rat anti-mouse Sca-1 (Abcam, dil. 1∶50) and mouse anti-mouse CD45 (eBioscence, dil. 1∶50). Secondary antibodies used were Alexa Fluor goat anti-mouse 594 nm (Invitrogen, dil. 1∶200), Alexa Fluor chicken anti-rabbit 594 nm (Invitrogen, dil. 1∶200) and Alexa Fluor goat anti-rat 568 nm (Invitrogen, dil. 1∶200). Immunostained samples were viewed with an Olympus IX71 inverted microscope.

### Immunocytochemistry on Cultured Satellite Cells

SCs clonally cultured for 5 days in 96-wells plate with 20% or 2% of O_2_ were rinsed in PBS and permeabilized with Triton X-100 0.3% in PBS for 10 minutes. Non-specific interactions were blocked with 10% HS for 30 minutes. After washing in PBS, cells were incubated with primary antibodies 1 hour at 37°C or overnight at 4°C. They were then washed and incubated with labeled secondary antibodies for 1 hour at 37°C. Nuclei were counterstained with DAPI. The following primary antibodies were used: rabbit anti-mouse Myf5 (Santa Cruz, dilution 1∶100), rabbit anti-mouse MyoD (Santa Cruz, dil. 1∶100), rat anti-mouse CD34 (GeneTex, dil. 1∶100), mouse anti-mouse α7integrin (dil. 1∶100), rat anti-mouse CD31 (eBioscence, dil. 1∶50) and mouse anti-mouse Desmin (Sigma-Aldrich, dil. 1∶500). Secondary antibodies used were Alexa Fluor goat anti-mouse 594 nm (Invitrogen, dil. 1∶200), Alexa Fluor chicken anti-rabbit 594 nm (Invitrogen, dil. 1∶200) and Alexa Fluor goat anti-rat 568 nm (Invitrogen, dil. 1∶200). Immunostained samples were viewed with a Leica B5000 microscope. The number of myotubes/clone was determined as the ratio between the total number of Desmin+myotubes observed and the total number of analysed clones.

### RNA Extraction and Amplification

Total RNA was extracted from single clones using RNeasy Micro Kit (Qiagen) following the manufacturer’s instructions. In order to obtain a sufficient amount of cDNA for the RealTime PCR experiments, purified RNA samples were amplified using TransPlex Complete Whole Transcriptome Amplification Kit (Sigma-Aldrich) in accordance with the manufacturer’s instructions [27]. Resulting cDNA has been quantified with a Nanodrop ND-2000 Spectrophotometer (Thermo Scientific) and we obtained an average of about 2 µg of cDNA from each clone.

### RealTime PCR

Quantitative RealTime PCR reactions have been carried out in triplicate with Lc FastStart Dna Master^PLUS^ SYBR (Roche) in a LightCycler II instrument (Roche), using 2 ng of amplified cDNA and 2 µL of a 3 µM mix of specific forward and reverse primers. Results have been expressed in arbitrary units as the ratio of target gene mRNA content to housekeeping gene mRNA content, further normalized for the number of cells in each considered clone, in order to obtain a “single cell equivalent” quantification. *β2microglobulin* was used as housekeeping gene. Primer sequences are reported in Table S1.

### *In vivo* Transplantation of Single Clones

SC clones were transplanted *in vivo* in a murine muscle injury model. *Tibialis anterior* (TA) of C57BL/6J mice were injected locally with 30 µL of cardiotoxin (*Naja nigricollis* snake venom, Latoxan) 10 µM. Single GFP+LPC and HPC were detached at 5 days of culture with 0.05% trypsin solution (GIBCO-Invitrogen). Single clones were re-suspended in 30 µL of PBS and injected separately 48 hours after injury in TA damaged muscles. Transplanted animals were sacrificed after 4 weeks, TA muscles were collected and fixed in PFA 4% for 1 hour at 4°C. Muscles were dehydrated at 4°C with a sucrose gradient method: sucrose 10% in PBS for 30 minutes, 15% for 30 minutes and 30% overnight. Then muscles were frozen in isopentane cooled in liquid nitrogen, and subsequently processed using a cryostat (Leica) to produce transverse sections for histochemistry and immunostaining.

#### Histochemistry

Haematoxylin and Eosin staining (Bio-Optica for rapid frozen section) of transplanted muscle was performed. Muscle sections were put in reagent A for 45–60 seconds and rinsed in tap water, then were dipped in Buffer solution and rinsed again in tap water. Slides were put in reagent C for 30 seconds and dipped first in ethanol 95%, then in absolute ethanol and finally in xylene. Sections were mounted in synthetic mounting medium.

#### Immunohistochemistry

GFP+single clone-transplanted-TA sections were rinsed in PBS and permeabilized with Triton X-100 0.5% in PBS for 10 minutes. Non specific interactions were blocked with 10% HS for 15 minutes at room temperature. After washing, slides were incubated with primary antibody rabbit anti-mouse Laminin (Sigma-Aldrich, dil. 1∶100) for 75 minutes at 37°C. They were then washed and incubated with labeled secondary Alexa Fluor chicken anti-rabbit 594 (Invitrogen, dil. 1∶200) for 1 hour at 37°C. After washing, sections were incubated with rabbit anti-Green Fluorescent Protein Alexa Fluor 488 Conjugate (Invitrogen, dil. 1∶200) for 1 hour to enhance GFP fluorescence signal. Nuclei were counterstained with fluorescent mounting medium plus 100 ng/mL DAPI.

For immunofluorescence and Engraftment Index (EI) analyses, 10 random fields for each analyzed muscle were acquired with an inverted microscope (Olympus IX71) at 20X magnification. Pictures were analyzed with Photoshop and ImageJ systems to count GFP+and centrally nucleated fibers. Random fields were collected from serial sections of the whole transplanted muscle to obtain wide representative sampling from different (apical, median and caudal) levels and areas. EI has been determined for each transplanted clone, dividing the corresponding percentage of GFP+fibers achieved for the number of GFP+transplanted cells (raw data in Table S2).

#### DNA extraction and PCR analysis

DNA from transplanted TA was extracted with a DNeasy Blood & Tissue kit (QIAGEN GmbH) and then quantified with a ND-1000 spectrophotometer (Thermo Scientific). Genomic DNA samples extracted from C57BL/6-(ACTB-EGFP)/J and WT mice organs were respectively used as positive and negative controls for the amplification of the *GFP* gene. PCR reactions for the *TERT* and *GFP* genes were carried out as previously described [28], primers sequences are reported in Table S1.

### Statistical Analysis

A model based cluster analysis with normal mixture modelling was applied [29] to identify the groups with the number of cells *per* clone. The number of groups and their classification were estimated with the algorithm mclust using R software.

Values are reported as means ± SEM. Significant differences were determined using one-way ANOVA and Student’s t-test when required, with * = p<0.05, ** = p<0.01 and *** = p<0.001.

## Results

### Hypoxia Increased Clonal SCs Proliferation

Individual myofibers were isolated from three month-old C57BL/6J mice hindlimb skeletal muscles after enzymatic digestion. Fiber-associated SCs were mechanically extracted (stripping) for clonal cultures (Fig. 1A). Each individual myofiber contains a few tens of SCs, and some hundreds of myofibers were collected to obtain several SC clone multiwell plates (a number between 12 and 20, 96-well plates were used for each experiment). Citospun were performed immediately after the stripping passage to investigate the myogenic phenotype of the isolated population (Fig. S1). Almost all the cells expressed Pax7 and α7integrin (94% and 96% respectively), which characterise quiescent SCs. Citospuned cells were also highly positive for CD34 membrane protein (88%). However, stripped SCs showed low percentages of MRFs expression: 13% of cells were positive for MyoD and 32% for Myf5. Finally, contaminant non-myogenic cells were not found in the freshly isolated population, as demonstrated by Sca1, CD31 and CD45 stainings.

Cells were subsequently seeded in proliferative medium and cultured in presence of high (20%–normoxia condition) or low (2% - hypoxia condition) oxygen levels. Initially, clones were observed and followed from day 2 to day 10 after seeding and a kinetic growth curve was made for each clone. The highest number of cell divisions was achieved at day 5 of culture for both oxygen conditions where clones reached the best ratio cells *per* clone. At day 10, indeed, cell proliferation tended to slow down with the formation of rare myotubes (data not shown). For this reason day 5 was chosen as the time point for further analyses. The number of clones after 5 days of culture was significantly higher in hypoxia condition (Fig. 1B), highlighting improved cell survival and growth at low oxygen. In addition, at this time of culture the number of cells *per* clone showed a bi-modal distribution. In fact, through a model based cluster analysis with normal mixture modelling of the distribution of cell number *per* clone, we identified two subpopulations with different proliferation rate as previously reported in rat [23]: Low Proliferative Clones (LPC) and High Proliferative Clones (HPC) (Fig. 1D). LPC and HPC showed i) homogeneous round and small spindle shape and ii) growth by uniform distribution without cluster formation (Fig. 1B) both at normoxia and hypoxia conditions. However, both LPC and HPC grew better at 2% than 20% O_2_ and therefore the cut-off value between the two subgroups was always twice in the hypoxia in respect to the normoxia condition (Fig. 1D and H). Furthermore, the significant difference between the number of cells *per* clone of LPC and HPC (Fig. 1E and I) not only confirmed their differences in proliferative abilities, but also highlighted the proliferation enhancement by hypoxia. ATP quantification underlined similar differences, revealing higher ATP levels in HPC than LPC (Fig. 1G). The amount of ATP, which signals the presence of metabolically active cells, is directly proportional to the number of cells present in culture. Moreover, ATP detection, both in LPC and HPC, was also significatively higher in hypoxia in respect to normoxia (Fig. 1M). In theory, the lower number of cells displayed by LPC compared to HPC could be the consequence of both/either cell death and/or proliferation. However, TUNEL assay performed after 5 days of culture was always negative, both in normoxia and hypoxia conditions for both clones (data not shown). Intriguingly, the two subgroups appeared in each experiment in similar proportions; in particular, about 30% of the total amount of clones were HPC, whereas LPC were more representative (about 70%) (Fig. 1F and L). This fixed proportion was very similar between the normoxia and hypoxia culture conditions and confirmed the previous observation in rat SCs [23]. Having revealed above that 98% of freshly isolated cells were Pax7+, non-SC contamination would not explain the presence of 30% cells belonging to HPC. Nevertheless, as in control experiments, the clonal subpopulations’ appearance was also validated by means of an alternative SC isolation protocol. Hindlimb skeletal muscles were subjected to enzymatic digestion. The resulting cell population FACS depleted for CD45, CD31 and Sca1, sorted for the expression of SM/C-2.6, (Fig. S2A), and cloned, revealed similar morphology and, most importantly, the same distribution in LPC and HPC as in stripped SC cultures (Fig. S2B and C). Together, these findings highlighted the higher proliferation profile of HPC than LPC, revealing proliferation heterogeneity within the SC population. These two subgroups were detectable both in normoxia and hypoxia, but with a clear proliferation enhancement in the latter. Thus, low oxygen increased SC clone proliferation maintaining their heterogeneity.

### LPC and HPC Differences were Maintained at Different Oxygen Concentrations

At day 5 of culture, the expression of several markers was analyzed using immunocytochemistry to understand whether the two subpopulations showed different expression signatures and how hypoxia could affect it. Alpha7integrin marker was completely maintained in both LPC and HPC (100%) during the culture period, both under normoxia and hypoxia (Fig. 2A and B). A higher proportion of cells expressed MyoD in LPC than in HPC and this difference was maintained at different oxygen concentrations (Fig. 2C and D). Similar results were observed when *MyoD* gene expression was evaluated at single cell level using RealTime PCR (Fig. 2M). In accordance, expression of Myf5 was higher in LPC than in HPC, but the marker reached a significant difference only in hypoxia condition, both at protein and gene expression level (Fig. 2E, F and N). Moreover, CD34, a typical quiescence SC marker that rapidly decreases after cell activation, showed significantly higher expression both at 20% and 2% O_2_ on LPC (56% and 55% respectively) when compared to HPC (41% and 40% respectively; Fig. 2G and H). Besides, CD31 was not present at all (0%) in all the conditions confirming the absence of cells from other origin (Fig. 2I and L). Evaluation of *hif-1α* expression at single cell level with RealTime PCR showed a higher expression of the factor in LPC compared to HPC, both under normoxia or hypoxia conditions (Fig. 2O). When cultured on proliferative medium, several desmin+myotubes were observed both in LPC and HPC clones. Intriguingly, low oxygen cultivation triggered myotube generation in all the clones (Fig. 3; ANOVA analysis showed p<0.05 among all the groups; Student t-test showed p<0.05 for both LPC and HPC hypoxia cultured clones compared to both normoxia cultured LPC and HPC). While, conversely, when adipogenic differentiation was considered, rare adipocytes were observed exclusively in HPC expanded in hypoxia and differentiated in adipogenic media in normoxia (Fig. S3).

### Hypoxia Culture Condition Induced Expansion of LPC Maintaining Myogenic Engraftment Potential *in vivo*

To test whether LPC and HPC possessed different muscle regeneration ability, C57BL/6J cardiotoxin damaged mice were transplanted with single GFP+clones cultured for 5 days under normoxia or hypoxia (Fig. 4A and Table S2). After 30 days the mice were sacrificed and transplanted TA were analysed for engraftment and muscle regeneration. Cell engraftment was confirmed by PCR (Fig. 4H). Single clones were able to generate GFP+fibers (Fig. 4B and Table S2), and LPC showed the greatest myogenic potential at single cell level. GFP signal was determined through staining with anti-GFP antibody. GFP+and GFP- fibers were counted in each random field and the percentage of GFP+fibers in respect to the total number of fibers was calculated. The percentage of GFP+fibers *per* transplanted TA (Table S2) was determined as the average of the percentages of GFP+fibers *per* field (the variance plotted in Fig. 4D and 4E was calculated on the basis of results derived from all the transplanted animals). In particular, GFP+fibers from normoxia cultured single LPC showed a higher trend in forming GFP+fibers than HPC (Fig. 4D), whereas hypoxia cultured LPC treated muscles possessed approximately twice GFP+fibers than the HPC treated (Fig. 4E). A similar trend was maintained when the number of centrally nucleated fibers was evaluated (Fig. 4F and G). Interestingly, hypoxia cultured clone transplantation led to a higher amount of GFP+centrally nucleated fibers (Fig. 4G right), highlighting a wider transplanted cell involvement in the regeneration process. However, quantifications of GFP+fibers and GFP+centrally nucleated fibers in transplanted muscles showed no statistically significant differences among the groups. This could be ascribed to the fact that fiber percentages do not take into account the number of transplanted cells *per* TA (Table S2). Starting from this observation, the Engraftment Index (EI) calculated as the proportion of GFP+fibers *per* number of GFP+transplanted cells, revealed that LPC cultured in normoxia and hypoxia conditions gave a significantly higher (5 fold) EI in respect to HPC (Fig. 5A and B). Therefore, (i) LPC cultured 5 days at 2% O_2_ grew efficiently allowing the transplantation of more cells *per* clone when compared to LPC normoxia cultured; and (ii) one single cell of LPC cultured both in normoxia and hypoxia possessed the same EI; in our setting, isolation and expansion of LPC at 2% O_2_ was the most efficient way to culture mouse SCs (Fig. 5B).

## Discussion

SCs are a population of stem cells, expressing Pax7 and responsible for postnatal skeletal muscle growth and repair. Although several cell types can contribute to muscle regeneration [30]–[36], SCs play a unique role in maintaining and replacing the muscle stem cell niche [37]–[40]. As a consequence, freshly isolated SCs showed the greatest regenerative potential when injected in a damaged muscle. However, this potential diminished *in vitro* even after a limited expansion [2], [41], [42]. This so far has restricted their clinical application and, despite various studies which have focused on mimicking SC niche conditions [43], [44], it is still a matter of debate which culture condition may favour the maintenance of stemness of SCs during expansion. Moreover, SCs are heterogeneous in their proliferative and myogenic potentials [23], [37], [45]–[54].

Here, for the first time, we found that the best muscle regenerative potential can be obtained when low proliferative satellite cell clones (LPC) were cultured in hypoxia (2% O_2_). Although they showed similar morphology, LPC and HPC were present in different proportions, with higher LPC development (∼70%) in respect to HPC (∼30%). This disproportion was fixed throughout all the experiments and reflected our previous work on rat SC clones [23]. We also excluded the possibility that intrinsic heterogeneity within SCs population was determined by the isolation method. Indeed, clone morphology and bi-modal distribution were confirmed among CD45−/CD31−/Sca1−/SM/C-2.6+cells. Several studies indicated that SCs constitute a population negative for the hematopoietic marker CD45, the endothelial marker CD31 and the Stem cell antigen 1 (Sca1), through flow cytometric analysis [25], [41], [55], [56]. After this Lineage depletion, different specific markers have been identified to isolate quiescent SCs: α7integrin, β1integrin, CD34, M-cadherin, c-met, CXCR4, caveolin-1, and syndecan-3 and -4 [5], [2], [25], [36], [45]–[50], [57]. In particular, SM/C-2.6 antibody permits detection of mononuclear cells residing beneath the basal lamina of myofibers that are positive for Pax7, M-cadherin, CD34 and CTR, negative for MyoD and Ki67, and which are capable of self-renewal, making this antibody suitable for quiescent SCs isolation [25], [58]–[60].

Interestingly, we found that LPC and HPC proportions were maintained even when cells were cultured at different oxygen concentrations. Oxygen may be indeed an important component of stem cell niche: the low oxygen condition, in fact, leads to increased proliferation and maintenance of the naïve state of stem cells [7], [10]. Here, the hypoxic conditions, while maintaining cell heterogeneity, displayed higher numbers of cells *per* clone and ATP quantity in respect to normoxia, as previously observed for other stem cells [7], [11]–[14], [61]. This has important implication in SC expansion and confirms previous work done on human SCs [7], [11]. Moreover, hypoxia influenced phenotype and differentiation capacity of SC clones. Hypoxia cultured HPC had the exclusive ability to differentiate into adipocytes when subsequently cultured in adipogenic conditions and normoxia. It still remains however, to understand if this could be an effect of a low oxygen environment [7] or whether HPC may represent *in vivo* a source of adipogenic cells within the skeletal muscle in pathological conditions [62]. We have shown that adipocytes can be derived from human SCs [46], while in rat, HPC, but not LPC, have been shown to be able to differentiate spontaneously into adipocytes [23].

It remains a matter of debate whether LPC or HPC may represent *in vivo* a distinct population with self-renewal characteristics. LPC have indeed shown a higher number of cells positive to Myf5 and CD34 which are commonly associated to quiescent or self-renewing SCs [63]–[65]. In particular, CD34 expression is extinguished shortly after the isolated fibers are placed in culture and become up-regulated only in SCs that revert back to a reserve state [66], [67]. Moreover, recently described slow-dividing SC subpopulations [37], [47], which show extensive contribution to muscle regeneration *in vivo*, resemble characteristics of LPC. On the other hand both Myf5 and CD34 are also expressed by HPC while LPC show higher presence of MyoD+cells, which is usually a characteristic of committed myogenic cells. Therefore, whether symmetric and asymmetric divisions, attributed to distinct subsets of SCs [37], [46], [47], [64], [68], belongs to LPC, HPC or both, still have to be investigated.

LPC and HPC differed also when expression of hypoxia-inducible factor-1 (HIF-1) was investigated. HIF-1 plays an essential role in oxygen homeostasis, regulating several genes involved in angiogenesis, glucose uptake, cell proliferation and survival [69]. At normoxia, this factor is constitutively expressed, but it is essentially regulated by ubiquitinylation and proteolytic degradation [70]. However, under severe hypoxia, the HIF-1α protein level increases by relaxing its degradation, and HIF-1α can form an active complex with HIF-1β.

Since hypoxia increased SC proliferation we determined the *HIF-1α* gene expression involvement in clones cultured under normoxia and hypoxia. Intriguingly, LPC showed higher *HIF-1α* expression at a single cell level than HPC, but similar expression levels between low or high oxygen conditions. This is in accordance with other studies, demonstrating that, in contrast to other tissues, skeletal muscle expresses high amounts of HIF-1α in normoxia as well as in hypoxia [71], [72]. It has been reported that the expression of HIF-1α synchronized with that of myogenic regulatory genes early during muscle regeneration. Furthermore, important observations from recent studies, described that HIF-1α protein expression decreases concomitantly with increased MyoD and myogenin protein expression after the induction of myogenic differentiation [73] and suggested that HIF-1α expression *in vitro* is regulated mainly at the protein level [74]. This fact could explain the absence of a statistical difference between normoxia and hypoxia cultured clones in terms of *HIF-1α* gene expression at 5 days of culture. Conversely, since HIF-1α was less expressed in HPC cells, the involvement of this hypoxia-inducible factor in satellite cell clone proliferation remains unclear and needs further investigation.

Similar to other studies [55], [56], [75] both mouse HPC and LPC can contribute *in vivo* to the SC pool when injected after injury. When single GFP+HPC and LPC clones were injected in C57BL/6J cardiotoxin damaged mice we observed skeletal muscle regeneration, which was more efficient with LPC cells. Despite the relatively low myotube formation displayed *in vitro*, single LPC transplantation effectively produced wider regenerating myofibers especially after hypoxic culture, supporting the idea that the population of slow-dividing SCs is capable of robust niche re-population and more efficient muscle regeneration than faster-dividing population [37], [47]. In particular, hypoxia increased both central- and peripheral-nucleated GFP+fibers in respect to normoxic conditions, suggesting that a low oxygen microenvironment could improve both SCs expansion and their *in vivo* active regeneration potential. We can speculate that culturing SCs in hypoxic conditions could improve the maintenance of a cell which may actively repopulate the *in vivo* SC niche and participate in new myofiber formation.

Extending these observations to a single cell level, the EI pointed out that hypoxia maintained the LPC single cell engraftment potential when transplanted *in vivo*. This implies that not only does a regenerative environment has a preferential effect for engraftment of slow-dividing cells [47], but this also confirms that the expansion achieved by low oxygen conditions do not impair the LPC *in vivo* potential.

In conclusion, our results show that 2% O_2_ allows for a better expansion of SCs and maintains their intrinsic myogenic potential for more efficient cell transplantation. This indicates the significant role hypoxia plays as a key component for SCs expansion. In particular, hypoxia conditions promoted LPC growth, SCs heterogeneity maintenance and *in vivo* myogenic potential preservation. Finally, our results provide insights into the heterogeneity in marker expression within the clonal population, highlighting possible differences in the activation state and self-renewal process. Future studies are required to better understand LPC and HPC properties in muscle homeostasis and regeneration.

## Supporting Information

Figure S1 **Freshly isolated SC marker expression characterization.** Freshly stripped SCs were characterized for their initial marker expression signature with immunofluorescence after citospun (in red the specific marker merged with DAPI; bar = 100 µm). Graphs indicated positive cell number averages (n = 8, mean±SEM). SCs displayed almost total positivity for Pax7, α7integrin and CD34, whilst they were negative for Sca1, CD31 and CD45. Respectively 13% and 32% of cells were positive for MyoD and Myf5.
(TIF)

Figure S2 **SCs sorted by SM/C-2.6 marker and clonal cultured, compared with stripped SCs.** SCs were isolated from hindlimb skeletal muscles after several enzymatic passages. After Lineage depletion (Lin-) for CD45, CD31 and Sca1 (A - left, 35.8% of total cell population was Lin-), SM/C-2.6+cells were sorted (A – right, 17.7% of Lin- cells, 2% of total cell population) and cultured at clonal density. After 5 days of culture, clone morphology appeared similar between SM/C-2.6+and stripped cells (B) and it was possible to distinguish between LPC and HPC in both the clonal cultures (B) (bar = 100 µm). The proportion of LPC versus HPC in SM/C-2.6+cell clones was similar to that obtained in stripped cells cultures (C – proportion between LPC and HPC in SM/C-2.6+clones, mean).
(TIF)

Figure S3 **Adipogenic differentiation of hypoxia cultured clones.** Clones cultured for 5 days in proliferative medium and subsequently for 10 days in adipogenic differentiation medium and normoxia. Adipocytes developed in HPC cultured initially in 2% O_2_ (right) and not in LPC (left) cultures. Insets with higher magnification of a single adipocyte with lipid vacuoles positive for Oil-Red-O specific staining (red, bar = 75 µm).
(TIF)

Table S1 **Primers list.**
(DOC)

Table S2 **Total single LPC and HPC transplanted clones and the corresponding percentage of GFP+fibers obtained.** Number of cells of each GFP+transplanted clones *per* injured TA and corresponding percentage of GFP+fibers achieved after 30 days from the transplantation.
(DOC)
