# Supplementary figures and images for: Hypoxia Increases Mouse Satellite Cell Clone Proliferation Maintaining both In Vitro and In Vivo Heterogeneity and Myogenic Potential
Source: PLoS One. 2012 Nov 16;7(11):e49860. doi: 10.1371/journal.pone.0049860 (PMC3500318; doi:10.1371/journal.pone.0049860)

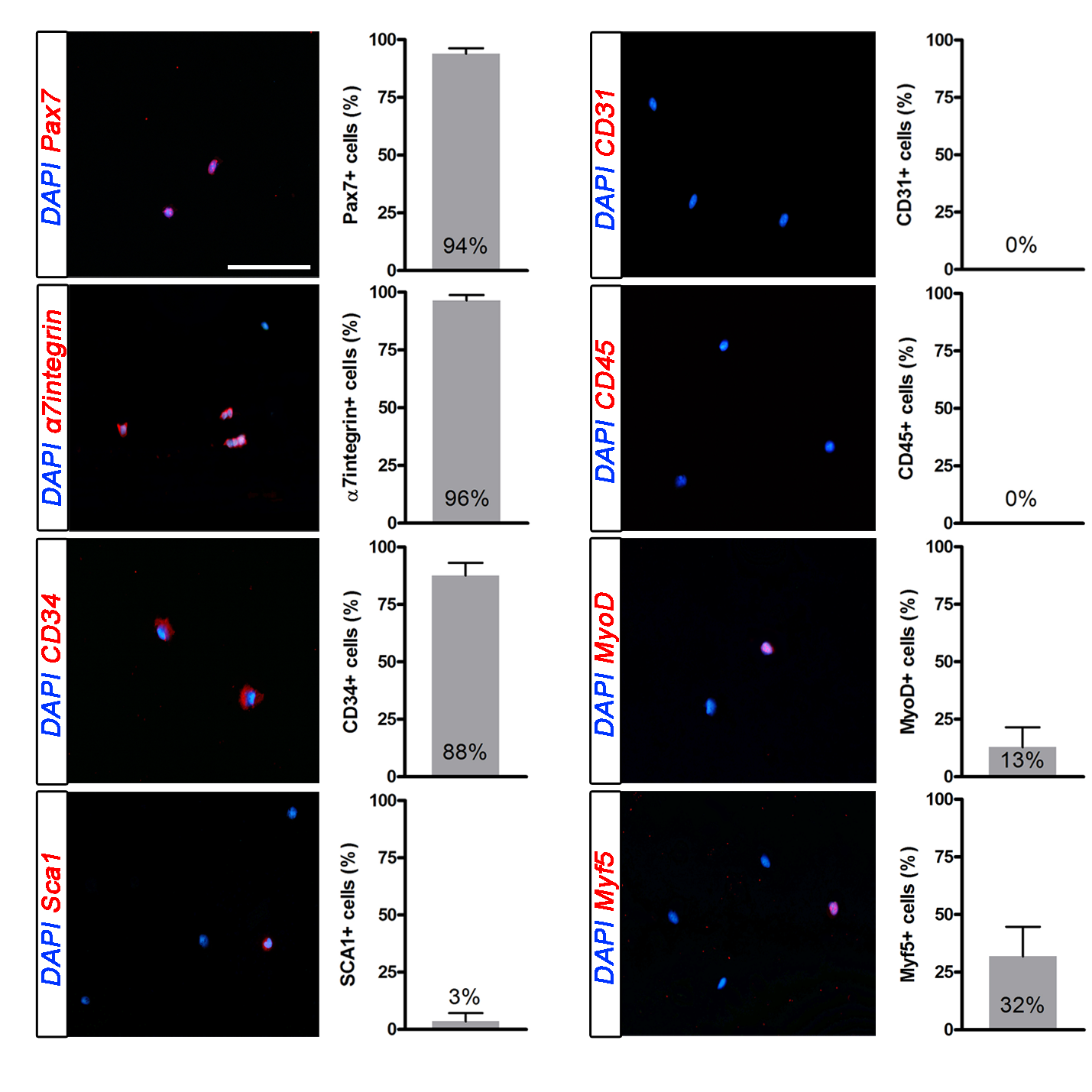

Supplement: Figure S1 — Freshly isolated SC marker expression characterization. Freshly stripped SCs were characterized for their initial marker expression signature with immunofluorescence after citospun (in red the specific marker merged with DAPI; bar = 100 µm). Graphs indicated positive cell number averages (n = 8, mean±SEM). SCs displayed almost total positivity for Pax7, α7integrin and CD34, whilst they were negative for Sca1, CD31 and CD45. Respectively 13% and 32% of cells were positive for MyoD and Myf5. (TIF) [file pone.0049860.s001.tif]

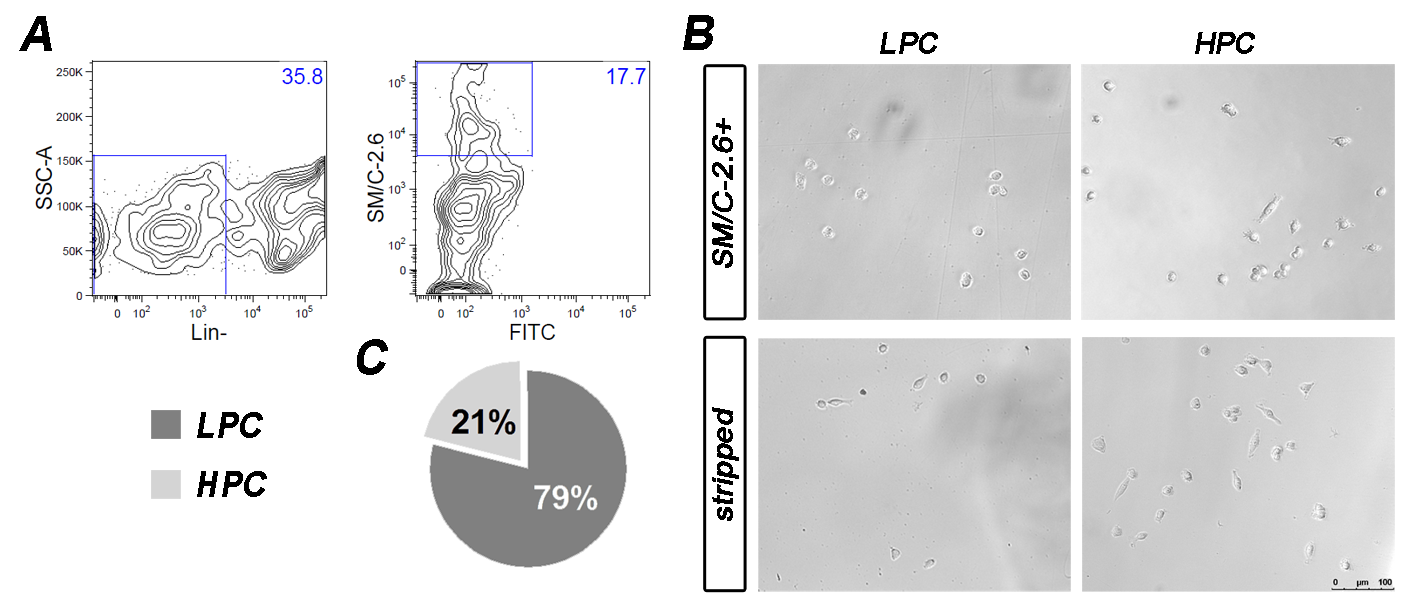

Supplement: Figure S2 — SCs sorted by SM/C-2.6 marker and clonal cultured, compared with stripped SCs. SCs were isolated from hindlimb skeletal muscles after several enzymatic passages. After Lineage depletion (Lin-) for CD45, CD31 and Sca1 (A - left, 35.8% of total cell population was Lin-), SM/C-2.6+cells were sorted (A – right, 17.7% of Lin- cells, 2% of total cell population) and cultured at clonal density. After 5 days of culture, clone morphology appeared similar between SM/C-2.6+and stripped cells (B) and it was possible to distinguish between LPC and HPC in both the clonal cultures (B) (bar = 100 µm). The proportion of LPC versus HPC in SM/C-2.6+cell clones was similar to that obtained in stripped cells cultures (C – proportion between LPC and HPC in SM/C-2.6+clones, mean). (TIF) [file pone.0049860.s002.tif]

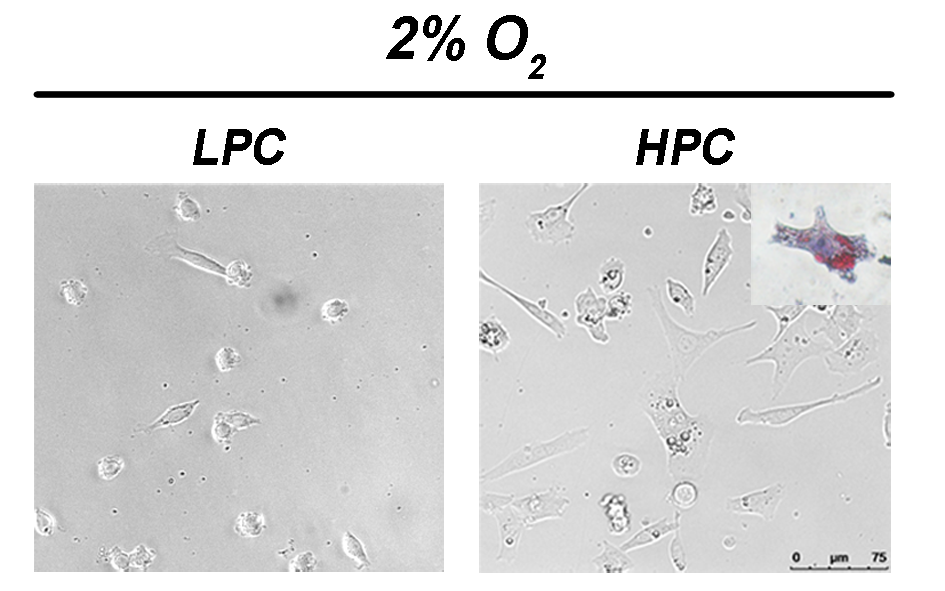

Supplement: Figure S3 — Adipogenic differentiation of hypoxia cultured clones. Clones cultured for 5 days in proliferative medium and subsequently for 10 days in adipogenic differentiation medium and normoxia. Adipocytes developed in HPC cultured initially in 2% O2 (right) and not in LPC (left) cultures. Insets with higher magnification of a single adipocyte with lipid vacuoles positive for Oil-Red-O specific staining (red, bar = 75 µm). (TIF) [file pone.0049860.s003.tif]
