# Supplementary material for: Hypoxia Increases Mouse Satellite Cell Clone Proliferation Maintaining both In Vitro and In Vivo Heterogeneity and Myogenic Potential
Source: PLoS One. 2012 Nov 16;7(11):e49860. doi: 10.1371/journal.pone.0049860 (PMC3500318; doi:10.1371/journal.pone.0049860)
Supplement: Table S1 — Primers list. (DOC) [file pone.0049860.s004.doc]

**Supporting table S1**

| |  |  |  | | --- | --- | --- | |  |  |  | |  |  |  | |  |  |  | |  |  |  | |  |  |  | |  |  |  | |  |  |
| --- | --- | --- | --- | --- | --- | --- | --- | --- | --- | --- | --- | --- | --- | --- | --- | --- | --- | --- | --- | --- | --- | --- | --- |
|  |  |  |
|  |  |  |
|  |  |  |
|  |  |  |
|  |  |  |
|  |  |  |

**Table S1.**

| **Name** | **Forward 5’ > 3’** | **Reverse 5’ > 3’** |
| --- | --- | --- |
| *MyoD* | CGCTCCAACTGCTCTGATG | TAGTAGGCGGTGTCGTAGCC |
| *Myf5* | ACAGCAGCTTTGACAGCATC | AAGCAATCCAAGCTGGACAC |
| *Hif-1α* | ACATCAAGTCAGCAACGTGG | CTGTGTCGACTGAGAAATGT |
| *β2microglobulin* | GCTTCAGTCGTCAGCATGG | CAGTTCAGTATGTTCGGCTTCC |
| *GFP* | TGAACCGCATCGAGCTGAAGGG | TCCAGCAGGACCATGTGATCGC |
| *gTERT* | ACCCACTATCCTTGTGGTGCATGA | AGATCGAGCAGCTGCAAGACCATA |
