# Supplementary material for: Hypoxia Increases Mouse Satellite Cell Clone Proliferation Maintaining both In Vitro and In Vivo Heterogeneity and Myogenic Potential
Source: PLoS One. 2012 Nov 16;7(11):e49860. doi: 10.1371/journal.pone.0049860 (PMC3500318; doi:10.1371/journal.pone.0049860)
Supplement: Table S2 — Total single LPC and HPC transplanted clones and the corresponding percentage of GFP+fibers obtained. Number of cells of each GFP+transplanted clones per injured TA and corresponding percentage of GFP+fibers achieved after 30 days from the transplantation. (DOC) [file pone.0049860.s005.doc]

**Supporting table S2**

Table S2.

| **20% O_2_** | | | | **2% O_2_** | | | |
| --- | --- | --- | --- | --- | --- | --- | --- |
| **LPC** | | **HPC** | | **LPC** | | **HPC** | |
| **# cells** | **% GFP+ fibers** | **# cells** | **% GFP+ fibers** | **# cells** | **% GFP+ fibers** | **# cells** | **% GFP+ fibers** |
| 13 | 22,0 | 34 | 2,4 | 26 | 14,9 | 76 | 10,9 |
| 12 | 20,6 | 32 | 0 | 26 | 18,0 | 86 | 0 |
| 12 | 9,7 | 48 | 0 | 24 | 16,8 | 65 | 6,3 |
| 12 | 11,2 | 66 | 18,0 | 25 | 12,1 | 58 | 32,8 |
| 14 | 0 | 33 | 5,9 | 32 | 20,1 | 76 | 7,7 |
| 13 | 7,2 | 24 | 16,4 | 24 | 28,0 | 86 | 0 |
| 16 | 0,5 | 30 | 6,4 | 24 | 35,6 | 87 | 31,7 |
| 12 | 0 | 30 | 2,4 | 28 | 31,5 | 85 | 19,5 |
| 12 | 44,1 | 51 | 16,3 | 24 | 41,2 |  |  |
| 11 | 5,5 | 40 | 21,2 | 19 | 0 |  |  |
| 13 | 18,1 |  |  | 26 | 19,1 |  |  |
| 15 | 11,1 |  |  |  |  |  |  |
